# Total Anthocyanin Content of Strawberry and the Profile Changes by Extraction Methods and Sample Processing

**DOI:** 10.3390/foods11081072

**Published:** 2022-04-07

**Authors:** Toktam Taghavi, Hiral Patel, Omololu E. Akande, Dominique Clark A. Galam

**Affiliations:** Agricultural Research Station, Virginia State University, Petersburg, VA 23806, USA; hpatel@vsu.edu (H.P.); oeakande@vsu.edu (O.E.A.); dgalam@vsu.edu (D.C.A.G.)

**Keywords:** flavonoids, organic solvent, methanol extract, chloroform extract, pH differential, ultra high performance liquid chromatography (UHPLC)

## Abstract

Anthocyanins are the primarily pigments in many flowers, vegetables, and fruits and play a critical role in human and plant health. They are polyphenolic pigments that are soluble in water and usually quantified by spectrophotometric methods. The two main methods that quantify anthocyanins are pH differential and organic solvent-based methods. Our hypothesis was that these methods extract different anthocyanin profiles. Therefore, this experiment was designed to identify anthocyanin profiles that are extracted by pH differential and organic solvent-based methods and observe their total anthocyanin content from strawberries. Six methods were tested in this experiment to quantify and profile anthocyanins in strawberry fruits by spectrophotometry and Ultra High Performance Liquid Chromatography (UHPLC) respectively. Four methods used organic solvents (methanol, and chloroform-methanol) in different combinations. The next two methods were pH differential and a combination of organic solvent and the pH differential method. The results suggest that acidified chloroform-methanol extracted the highest anthocyanin content compared to water-based solvents. Methanol-water based solvents also performed better than methanol alone, because both methanol and water may extract different profiles of anthocyanins. Water-based extracts had the greatest absorbance at a lower wavelength (498 nm), followed by methanol (508 nm), and chloroform (530 nm). Chloroform-methanol solvent with higher pH (3.0) extracted pelargonidin as the main anthocyanin, while methanol and water-based solvents (with lower pH 1.0–2.0) extracted delphinidin as their main anthocyanin as identified by UHPLC. Therefore, chloroform-methanol and methanol-water solvents were the best solvents for extracting anthocyanins from strawberries. Also, freeze-dried strawberries had higher anthocyanin contents compared to fresh or frozen samples.

## 1. Introduction

Anthocyanins are primarily found in fruits, vegetables, and flowers and provide health benefits to plants and human. Therefore, research into the cellular effects of anthocyanins has been on the rise. Strawberries are a rich source of anthocyanins and have attracted a lot of attention due to their health benefits [1,2]. Research has also been focused on the physiological functions of anthocyanins in strawberries. Therefore, identification of the extracted anthocyanins and their accurate quantification is of paramount importance [3].

Several methods have been developed for anthocyanin quantification. Initially, capillary electrophoresis and classic chromatographic methods such as paper and thin-layer chromatography were used. More recently, non-destructive methods such as spectral reflectance are being developed [4]. However, traditional analytical techniques are still widely used to measure the anthocyanin concentrations. More commonly, anthocyanins are quantified by spectrophotometric methods in plant tissues [5]. Two main spectroscopic methods are based on organic solvents and pH differential buffers that extract anthocyanins in an acidic environment [6].

Neff & Chory [7] used organic solvent-based (chloroform-methanol) for anthocyanin assessment from Arabidopsis leaf samples. Solovchenko et al. [5] also used a different ratio of methanol for an anthocyanin assessment of apple peel samples. They suggested adding chloroform would eliminate the impurities that interfere with light absorption. While the later group did not use water, Neff & Chory [7] added water to the extraction buffer.

The second spectrophotometric method is the pH differential method. In this method, the structural changes in anthocyanins in different samples’ pH is the base for rapid and accurate measurement of the total anthocyanins [8,9]. At a pH of 4.5 the anthocyanin is colorless, while at a pH of 1.0, the anthocyanin absorbs light very strongly between 460 and 550 nm. Thus, the absorbance at 520 nm is proportional to the concentration of pigment [10]. Gauche et al. [11] suggested combining the two methods by extracting anthocyanin with acidified methanol and reading absorbance by the pH differential method to remove light absorbing impurities.

Due to the complex chemistry of the pigment, an efficient extraction method suitable for the different food matrixes needs to be identified [12]. Different methods have been used by researches to extract anthocyanins from different fruit crops with unique matrixes. For example, both acidified methanol and pH differential buffers were used for strawberries. However, it is not clear which method is best suited for strawberries and there is no comparison between them on extracting different anthocyanin profiles [2,10,13,14].

Total anthocyanin content was affected by assessment methods and the extracted anthocyanins had different colors and profiles [15]. Also, different sample-to-buffer ratios were used for different assessment methods, and correction factors have been included in the formula to account for different ratios. However, there was evidence that the solvents were saturated and the absorbance was not within the linear range of the spectrophotometer. There are also concerns that different sample-to-buffer ratios may significantly affect total anthocyanin content. Therefore, errors may have been created in calculating the total anthocyanin content. In this experiment, one single sample-to-buffer ratio was used for all methods, which were in the linear range of the spectrophotometer (<1.4 AU) suggested by Lee et al. [10].

The objectives of this experiment were to compare the effect of (1) sample preparation methods (fresh, frozen, and freeze-dried puree) and (2) six anthocyanin assessment methods on anthocyanin yield and profile of strawberry fruits. Two improvements were also included in the methods. First, a bulk sample was used to eliminate variations among samples, and second, a uniform, low sample-to-buffer ratio was used to compare the results of different methods within the linear range of the spectrophotometer.

## 2. Materials and Methods

### 2.1. Chemicals

Cyanidin-3-O-glucoside chloride (C3G), pelargonin chloride (PG), potassium chloride, sodium acetate, methanol, hydrochloric acid, and chloroform, were purchased from Sigma (Saint Louis, MO, USA). Mega pure water was obtained from Thermo Scientific Barnstead Smart2Pure 3 LPH UV/UF system.

### 2.2. Instrumentation

The equipment and software used in this experiment included a Visionlite 5 software connected to a UV/Visible spectrophotometer (Genesys 150 UV-Vis), a refrigerated incubator, a shaker (all from Thermo Fisher Scientific, Waltham, MA, USA), and a blender (Magic Bullet 600-Watt).

### 2.3. Standard and Blank Preparation

A Cyanidin-3-Glucoside (C3G), and a pelargonin chloride (PG) standard solution was made by dissolving 4, and 0.67 mgL^−1^ respectively, in Methanol:HCl (0.1%) and storing at −25 °C until further use. Both were used as an internal standard for wavelength scanning spectrum of absorbance. Distilled water was used as blank in spectrophotometer for all methods, as absorbance of the buffer is nil at the measured wavelengths [8].

### 2.4. Sample Preparation

About 500 g of strawberry fruits were lyophilized and ground to a fine puree using the blender, creating a uniform representative sample. One-gram portions of the puree were weighed and placed in 50 mL tubes. The tubes were divided in three groups. The first group was used as fresh samples for the experiment. The second and third groups were frozen quickly (−30 °C) to reduce oxidative modifications. The third group was then freeze-dried (freeze-dried puree) at −80 °C (VirTis Freezemobile freeze dryers SP Scientific, Warminster, PA, USA). The second and third groups were used as frozen and freeze-dried samples respectively.

### 2.5. Anthocyanin Assessment Methods

For the extraction of anthocyanins in strawberries, a total of six methods were tested. The methods were slightly modified to keep a constant sample-to-buffer ratio of 1:20 in all methods. The three strawberry sample types (fresh, frozen and freeze-dried) used in all methods were from a uniform pureed sample. Therefore, differences in anthocyanin yield and absorbance are related to the ability of a specific method to extract different anthocyanin profile, prevent pigment degradation, and remove impurities that interfere with the light absorption.

The first method is the chloroform-methanol method, which was suggested by Solovchenko et al. [5] and was used with some modifications. Anthocyanins were extracted using acidified methanol and chloroform. Extracts were obtained by adding 20 mL of chloroform-methanol (2: 1 v/v, acidified with 0.1% HCl) to the strawberry samples.

In the second method (methanol method developed by Solovchenko et al. [5]), chloroform was deleted and 20 mL of methanol (acidified with 0.1% HCl) was added to the strawberry samples.

The third method is a methanol method suggested by Lindoo & Caldwell [6]. The methanol:water:concentrated HCl (80:20:1) was added to the strawberry samples to a final volume of 20 mL. For the first three methods, the homogenates were incubated at 4 °C in the dark in the shaker for 48 h. At the end of the incubation period, the homogenates of all three methods were centrifuged at 4 °C and 7000 rpm for 15 min. The supernatant was then removed and stored in 15 mL tubes at −30 °C. The absorbance of anthocyanin was measured by the spectrophotometer at 530 and 657 nm [6,16].

The fourth method (chloroform-methanol) was based on the Neff & Chory [7] suggestions. For this method 15 mL methanol, 10 mL water, 0.15 mL HCL were mixed as the buffer. Twenty ml of the buffer and 20 mL chloroform were added to the strawberry samples and then incubated at 4 °C for 48 h in the dark on a shaker and then centrifuged at 4 °C, 7000 rpm for 15 min. The supernatant was then removed and stored in a 15 mL tube at −30 °C. The absorbance (*A*) was measured at 530 and 657 nm. Anthocyanin concentration was calculated by the following formula [2] and was given as A/g fresh fruit tissue, where *TA* = total anthocyanin, *A* = absorbance at 530 and 657 nm, *V* = volume of extract (mL) and *M* = fresh mass of the sample (g).

$$TA=\frac{A_{530}-0.3\;A_{657}\times V}{M}$$

In the fifth method, the content of strawberry anthocyanin was measured by the pH differential method presented by Lee et al. [10]. The strawberry samples were mixed thoroughly with 20 mL buffer pH 1.0 (0.025 M potassium chloride) and pH 4.5 (0.4 M sodium acetate buffer) and then incubated for 20 min at room temperature and centrifuged at 4 °C and 7000 rpm for 15 min. The supernatant was then removed, and the absorbance was read at 520 and 700 nm. This following formula was used to calculate the anthocyanin concentration.

$$TA=\frac{A\times V}{M}$$

where *A* = (*A*_520_ nm − *A*_700_ nm) pH 1.0 − (*A*_520_ nm − *A*_700_ nm) pH 4.5; *V* = volume of extract (mL) and *M* = fresh mass of the sample (g).

In the sixth method, Gauche et al. [11], 20 mL of 80:20:1 (methanol:water:HCl) buffer were added to the strawberry samples and incubated in darkness for 48 h. The crude extract obtained was centrifuged at 4 °C and 7000 rpm for 15 min. The supernatant was removed (step 1), and half of the original volume (10 mL) was concentrated under vacuum (−10 Evaccume psi) at 35 °C overnight. The quantification of total anthocyanin content in the concentrated extract was measured by adding ten ml of methanol:water:concentrated HCl (80:20:1) to the vaccumed strawberry samples (step 2).

**Figure 1:**
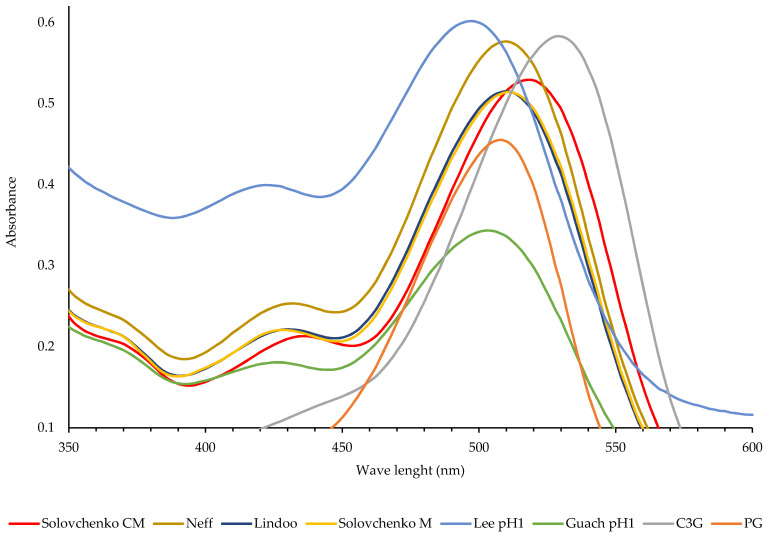
The absorption spectrum of fresh strawberry anthocyanins extracted from six different methods. The methods were: (1) methanol (M) method by Solovchenko et al. [5], (2) chloroform-methanol (CM) method by Solovchenko et al. [5], (3) methanol method by Lindoo and Caldwell [6], (4) chloroform-methanol method by Neff & Chory [7], (5) pH differential method (pH 1.0) by Lee et al. [10], and (6) combination of methanol and pH differential methods (step 2, pH 1.0) by Gauche et al. [11]. Cyanidin-3-O-glucoside (C3G) and pelargonin (PG) were used as the internal standards.

The tested methods used the same sample-to-buffer ratios (*w/v*), therefore, the formulas did not consider any correction factor for differences in the ratios and were used directly to calculate and compare the anthocyanin content of different methods. The first replicate was used to create the wavelength scanning spectrum of the absorbance. The spectrum absorbance was very similar for fresh vs frozen or freeze-dried samples, therefore, the spectrum data for only fresh samples were shown in Figure 1.

### 2.6. UHPLC Profiling of Individual Anthocyanins

Total anthocyanins in fresh, frozen and freeze-dried strawberry samples were extracted by six methods. The Lee and Gauche methods used two buffers (pH 1.0 and pH 4.5) and Gauche method had two-step extraction process. Samples from the six methods, after mixing with both buffers and completing both steps, were profiled by Ultra High Performance Liquid Chromatography (UHPLC), making the total number of extracted samples nine. Therefore, there was a combination of 27 (9 extracted samples × 3 sample types), however, the results of extracts with low pH buffers are presented. All extracted samples were centrifuged again at 12,000 rpm and 4 °C for 10 min, and the supernatants were transferred to vials for LC-MS analysis. The separation and identification of individual anthocyanins in each extraction method was performed by using Vanquish UHPLC combined with Q Exactive MS (Thermo) and screened with ESI-MS. The LC system is comprised of an ACQUITY UPLC HSS T3 (100 × 2.1 mm × 1.8 μm) with Vanquish UHPLC. The mobile phase is composed of solvent A (0.1% formic acid water) and solvent B (0.1% formic acid acetonitrile) with a gradient elution (0–2.0 min, 95% A; 2.0–15.0 min, 95–70% A; 15.0–15.1 min, 70–5% A; 15.1–20 min, 5% A; 20–20.1 min, 5–95% A; 20.1–26 min, 95% A). The flow rate of the mobile phase is 0.3 mL·min^−1^. The column temperature is maintained at 40 °C, and the sample manager temperature is set at 4 °C. Mass spectrometry parameters in ESI+ mode listed as follows: Heater Temp 350 °C; Sheath Gas Flow rate, 40 arb; Aux Gas Flow Rate, 10 arb; Sweep Gas Flow Rate, 0 arb; spray voltage, 3.0 KV; Capillary Temp, 320 °C.

The anthocyanins in the sample were identified by their retention times using standards for 103 anthocyanins (including but not limited to cyanidin-3-glucoside (C3G), malvidin-3-glucoside (M3G), pelargonidin-3-glucoside (P3G), and pelargonidin-3-5-diglucoside (P3-5DG), delphinidin-3-glucoside-chloride). The pH of the extracts were also measured before analyzing with UHPLC.

### 2.7. Experimental Design and Statistical Analysis

All the anthocyanin assessments methods had ten replicates and were repeated two times. The average data was used to calculate anthocyanin concentration. The anthocyanin concentrations of all the data were analyzed by Statistical Analysis Software (SAS Institute Inc. 2013. SAS/ACCESS^®^ 9.4, Cary, NC, USA) [17]. The experimental design was a completely randomized design. The first replicates was used to create wavelength scanning of absorbance spectrum for all different extracts.

## 3. Results

This paper compares organic solvents (methanol, chloroform-methanol), and pH differential methods for the extraction of anthocyanins in a bulk fruit sample of strawberries. The uniform bulk sample eliminates the sample differences and ensures yield variations, and absorbance spectrum reflect the extraction method efficacy to extract anthocyanins. The organic solvent and pH-differential-based methods were chosen because they are simple, accurate, and rapid methods for measuring monomeric anthocyanin content. The wavelength scanning spectrum of absorbance was also measured, and cyanidin-3-O-glucoside (C3G) and pelargonin (PG) were used as the internal standards, as the most common form of anthocyanins in nature, and strawberries respectively.

### 3.1. Total Anthocyanin Content

The chloroform-methanol methods suggested by Solovchenko et al. [5], and Neff & Chory [7] and the methanol method, Lindoo & Caldwell [6] extracted the highest total anthocyanin contents which were significantly higher than other methods. The other methanol method, Solovchenko et al., [5], was the second best method for extracting the highest amount of total anthocyanins. The combined method by Gauche et al., [11] extracted the lowest amount of anthocyanin and the pH differential method of Lee et al., [10] was slightly higher than the combined method (Table 1).

Because a uniform sample-to-buffer ratio was used for all methods, the higher anthocyanin content is not due to the lower sample-to-buffer ratio, as was hypothesized by authors, but rather the ability of the these methods to extract higher amounts of anthocyanins from strawberry puree.

Both chloroform-methanol methods suggested by Solovchenko et al. [5], and Neff & Chory [7] had similar buffer components, except that the Neff & Chory [7] method had water in its buffer, unlike the other method. Water caused the chloroform phase to separate during the centrifugation and anthocyanins stayed in the methanol:water phase.

The pH differential method, which is used extensively by the food industry, was the fastest method with only 20 min of incubation time. However, the anthocyanin concentration was lower than the methanol and chloroform-methanol methods (Table 1). A haze also formed that was not fully removed by centrifugation at 7000 rpm. The freeze thaw process of the extracts also increased the haze and reduced the absorbance (data not shown). Thus, this method can assess the anthocyanin content very quickly for a large number of samples, but is not recommended for storage of the extracts.

The combined method of Gauche et al. [11] had the lowest total anthocyanin content. This method has two steps, (1) extraction by methanol buffer and (2) assay with pH differential buffers, which creates a lengthy process with a higher chance of pigment degradation. For these two reasons, lower anthocyanin yield and longer processing time, it is not suitable for anthocyanin extraction in strawberries.

The sample type was also effective on total anthocyanin content. The freeze-dried samples released significantly higher amounts of anthocyanin into the extraction buffer compared to the frozen and fresh samples (Table 1).

### 3.2. Anthocyanin Absorption Spectrum

The standard anthocyanins, PG, and C3G spectrum had the maximum absorbance (λ_max_) at 508 and 530 nm respectively. The pH differential method by Lee et al., [10] had the lowest λ_max_ at 498 nm, followed by the combined method of Gauche et al. [11] at 504 nm, methanol methods suggested by Lindoo and Caldwell [6] and Solovchenko et al. [5] and the chloroform-methanol method suggested by Neff and Chory [7] at 510 nm, and the chloroform-methanol method by Solovchenko et al. [5] at 518 nm. The data suggest anthocyanins in water had lower λ_max_, and methanol and chloroform shifted the peak to the right, with chloroform forcing a stronger shift. The values for λ_max_, in chloroform were about 8 nm towards longer wavelengths, and in water (pH buffer) about 12 nm towards shorter wavelengths, than those given in methanol (Figure 1).

The pH differential method by Lee et al. [10] had a wider absorbance range (between 200–500 nm) than any other method, which is related to water-soluble phenolic or flavor compounds extracted by pH buffers. In all extracts, a peak between 300–320 nm appeared which does not interfere with anthocyanins (data not shown), and may be related to the flavor compounds or phenols, as prevalent in strawberries.

### 3.3. UHPLC Profiling of Individual Anthocyanins

Anthocyanins of the extracted samples were analyzed by the UHPLC-MS system and compared against 103 anthocyanin standards. The relative retention times (RT) have been detected specifically at the expected retention times [18]. About 50 anthocyanins (out of 103) were detected in samples by UHPLC-MS analysis (Table 2). Delphinidin (C_15_H_11_O_7_), cyanidin (C_15_H_11_O_6_), pelargonidin-3-o-sophoroside-5-o-glucoside (C_33_H_41_O_20_), and pelargonidin (C_15_H_11_O_5_), were the most frequent anthocyanins extracted by different methods.

The chloroform-methanol method of Solovchenko et al. [5] was the only method that extracted pelargonidin (C_15_H_11_O_5_), and petunidin (C_16_H_13_O_7_) as the main anthocyanins in its profile. All other methods extracted delphinidin (C_15_H_11_O_7_) as their main anthocyanin.

All methods extracted delphinidin, and cyanidin. However, cyanidin was not detected in frozen samples in chloroform-methanol method suggested by Neff and Chory [7] method. Pelargonidin-3-O-sophoroside-5-O-glucoside was identified in all methods except frozen and freeze-dried samples in methanol method of Solovchenko et al. [5] and freeze-dried samples in chloroform-methanol method of Neff and Chory [7]. Also, pelargonidin was identified in all methods except fresh and frozen samples in methanol method of Solovchenko et al. [5] and frozen and freeze-dried samples in pH differential method of Lee et al., [10] (Table 2).

Malvidin-3-o-arabinoside (C_22_H_23_O_11_) and peonidin-3-o-galactoside (C_22_H_23_O_11_) were prevalent anthocyanins identified in only about one third of extracts. From the 50 identified compounds, 15 of them were only detected in one extract type (Table 2).

The differences in anthocyanins identified in different extraction methods could lie in the solvent type and the pH differences. Chloroform-methanol method of Solovchenko et al. [5] was the only method with pH of 3.0 or more, while the other methods had pH between 1.0–2.0 (Table 2). Pelargonidin (C_15_H_11_O_5_) was the main anthocyanin in pH 3.0, while delphinidin (C_15_H_11_O_7_) was the main one in pH of about 1.0.

## 4. Discussion

Anthocyanin content of foods and produce changes by altering extraction conditions [19]. Due to the complex chemistry of the pigment, the extraction method of different food matrixes needs to be optimized [12]. The extraction of anthocyanins is usually accomplished by macerating, soaking and subsequently extracting anthocyanins with a solvent [19].

The recovery rate of anthoacyanins is also affected by method of extraction. The rough conditions of extraction in the ultrasound and microwave-assisted methods lead to a lower recovery rate compared to maceration with organic solvents in red grapes [18], and therefore maceration was used in this experiment.

Barnes et al. [20] tested a variety of homogenization procedures in blueberries using macerating in a mortar and pestle, grinding in a coffee grinder, and lyophilization in a freeze-drier. In blueberries, lyophilization did not affect the total anthocyanin content [20] but increased the reproducibility of the results [21] by eliminating the variability among individual blueberries, and suggested the need for bulk sample homogenization. To eliminate variability among samples, strawberries were bulk-homogenized in this experiment.

In other experiments with wheatgrass [22] and onion [23], the anthocyanin yield increased in freeze-dried samples, mainly due to the fact that ruptured plant cells during freeze-drying release phenolic compounds. The result emphasizes the importance of method optimization for different food matrixes and extraction methods, as explained by Silva et al. [12], and Taghavi et al. [15]. Our data also confirmed that freeze-dried samples had higher anthocyanin contents primarily due to ruptured cells and released anthocyanins in the extraction buffer.

Several other parameters affect the anthocyanin yield, such as temperature, incubation time, sample-to-buffer ratio, and solvent type [19]. The same temperature was used for all the tested methods. All methods used 48 h incubation time uniformly, except pH differential method, which had 20 min incubation time as longer incubation time was not recommended [10].

### 4.1. Sample to Buffer Ratio

Karaaslan & Yaman [19] found that the best extraction conditions in strawberries were to have a sample-to-buffer ratio (v:w) of 1:1, with methanol as solvent, and a 30-min extraction time [19]. However, Ghassempour et al. [18] concluded that a lower sample to buffer ratio (0.06 = 1/15) increased total anthocyanin content from grape skin tested by response surface methodology. This has been attributed to the limited capacity of extraction buffer. Their suggested ratio is very similar to what we used in this experiment (0.05 = 1/20). We also tested a sample-to-buffer ratio of 0.03 (1/30) for chloroform-methanol by Solovchenko et al. [5] method (data not presented) which increased anthocyanin yield significantly and had an absorbance below 0.8. Although Rousseau [24] believed too much solvent will excessively dilute anthocyanin concentrations in the final extract, we concluded that a lower sample-to-buffer ratio increases anthocyanin yield. Our data suggest that absorbance lower than 0.8 (between 0.2–0.8) represents the best sample-to-buffer ratio and higher absorbance reflects the saturated solvent. This absorbance range does not agree with what Lee et al. [10] suggested. They mentioned absorbance between 0.2–1.4 were in the linear range of spectrophotometer. However, in our experiment, using absorbance above 0.8 led to significantly lower anthocyanin content from the same sample. Because different sample-to-buffer ratios affect the results, only one ratio (0.05 = 1/20) was used in this experiment to eliminate the variability that the sample-to-buffer ratio may create.

### 4.2. Solvent Type

Boeing et al. [25] studied the solvent type effect on phenolic compound extraction of berries. They used three different organic solvent ratios (of methanol, ethanol, and acetone) and distilled water for extraction. The highest anthocyanin content was measured in methanol/water/acetic acid (70:29.5:0.5, v/v/v), which is very similar to our methanol method by Lindoo and Caldwell [6] with methanol:water:acetic acid ratio of 80:20:1 (v/v/v).

Acidified methanol has been suggested to extract the highest amount of anthocyanins, thus serves as the best and most commonly used anthocyanin extraction solvent [19,26]. Metivier et al. [27] also confirmed that acidified methanol was the most effective solvent for anthocyanin extraction from grape pomace. Acidified methanol was 73% more effective than water alone and 20% more effective than ethanol. Awika et al. [28] found that acidified methanol (0.1% HCl) was extracted twice the amount of anthocyanins from black sorghum compared to 70% aqueous acetone. The aqueous acetone extract modified the structure of anthocyanins by oxidative addition, which made them undetectable by HPLC [28]. Therefore, we used acidified methanol in this experiment. Acidified solutions help anthocyanins to penetrate through the cell membrane and be released into the extraction buffer. Low concentrations of HCl or other strong acids, are recommended [26] and 0.1% HCl were used in this experiment.

The pH of the solution may also affect the extraction yield and the stability of anthocyanins in the solution. A pH of 1 to 3 prevents the formation of the colorless anthocyanins that are of hemiketal form.

### 4.3. Total Anthocyanin Content

The higher amount of anthocyanins extracted by chloroform:methanol methods suggested by Neff and Chory [7], and Solovchenko et al. [5] could be due to the presence of chloroform in their buffers. Solovchenko et al. [5] claimed that chloroform reduces light-absorbing impurities.

The previous experiment done by Taghavi et al. [15] has shown that the chloroform-methanol and methanol methods suggested by Neff and Chory [7], Solovchenko et al. [5], and Lindoo and Caldwell [6] were the most efficient methods for anthocyanin extraction of frozen strawberry fruits. This experiment confirms the previous results. The reason for lower anthocyanin content in the methanol method by Solovchenko et al. [5] compared to other methanol or chloroform-methanol methods is not clear. The only explanation is mainly a lack of water in the extraction buffer that interferes with the anthocyanin extraction method. Also, a uniform sample-to-buffer ratio was used in this experiment to eliminate differences.

In conclusion, chloroform-methanol and methanol methods by Neff and Chory [7]; Solovchenko et al. [5], and Lindoo and Caldwell [6] extracted the highest amounts of anthocyanins, especially when water was eliminated by drying the samples (freeze-dried) or otherwise eliminating it from the buffer.

Solovchenko et al. [5] mentioned that extracts of methanol were turbid in apple peel extract, and chloroform eliminated the turbidity. However, the methanol fruit extracts in strawberry were clear and no turbidity was observed, and the addition of chloroform did not affect turbidity, but increased total anthocyanin content significantly compared with other methods.

### 4.4. Anthocyanin Absorption Spectrum

Generally, the solvent in which the anthocyanins are dissolved is important because it has an impact on their quaternary structures, which affects the color of any of the primary, secondary, or tertiary structures of anthocyanins and subsequently the maximum absornace (λ_max_). Ahmadiani et al. [29] revealed that anthocyanins had higher λ_max_ and molar absorption in methanol compared to the aqueous solvent resulting in a more violet/red-to-pink color with higher color intensities, while they shift towards a more yellowish hue in water. Harborne [30] stated that, the values for λ_max_, in ethanol are about 10 nm towards longer wavelengths and in water about 15 nm towards shorter wavelengths than those given in methanol.

The data confirm the reports, as the peak for the pH differential method by Lee et al. [10] appeared at a shorter wavelength (498 nm), the methanol methods by Lindoo and Caldwell 960; and Solovchenko et al. [5] at medium (510 nm), and the chloroform-methanol method appeared at the longest wavelengths (518 nm). In general, the values for λ_max_, in chloroform were about 8 nm towards longer wavelengths and in water about 12 nm towards shorter wavelengths than those given in methanol.

Our data support the previous report, except in the case of the chloroform-methanol method by Neff and Chory [7], which had the same λ_max_, as methanol methods. The reason is that, although we categorized the Neff and Chory [7] as a chloroform-methanol method, the chloroform was separated during centrifugation, therefore, the peak appeared at the same wavelength as the methanol methods.

### 4.5. UHPLC Profiling of Individual Anthocyanins

Health-conscious consumers are showing increasing interest in food products containing natural colorants due to their potential health benefits and the safety concerns regarding their synthetic counterparts. The trend towards high antioxidant-containing foods have switched the focus of plant breeding programs towards creating varieties with increased contents of health-related bioactive compounds [31].

The anthocyanin composition in strawberries has been studied by various researchers, but is still not fully characterized [32]. More than 25 different anthocyanins have been reported in strawberry cultivars and selections. Most of the strawberry anthocyanins are derivatives of pelargonidin and cyanidin. Dzhanfezova et al. [31] identified pelargonidin 3-O-glucoside as the main anthocyanin in 18 strawberry cultivars or selections. The other major three being pelargonidin 3-O-glucoside, pelargonidin 3-O-rutinoside and cyanidin 3-O-glucoside. These three compounds represented more than 95% of total anthocyanins in strawberries [32].

Donno et al. [33] identified four major anthocyanins: cyanidin-3-glucoside, pelargonidin-3-glucoside, pelargonidin-3-rutinoside, and pelargonidin-3-acetylglucoside in five strawberry selections by HPLC-DAD/MS. Karaaslan & Yaman [19] determined that the dominant anthocyanin for strawberry was pelargonidin-3-o-glucoside. Kawanobu et al. [34] reported that major anthocyanins in strawberry cultivars they studied were cyanidin-3-glucoside, pelargonidin-3-glucoside, and pelargonidin-3-malonylglucoside [19].

Pelargonidin-3-O-glucoside was about ten times higher than cyanidin-3-o-glucoside and the content of delphinidin-3-o-glucoside and malvidin-3-o-glucoside was less than detection limit in HPLC-ESI (electrospray ionization-MS [19]).

Kelebek and Selli [35] determined the anthocyanins of three strawberry cultivars to be cyanidin-3-glucoside, cyanidin-3-rutinoside, pelargonidin-3-glucoside, pelargonidin-3-rutinoside, pelargonidin-3-malonyl-glucoside, and pelargonidin-3-acetyl-glucoside for three strawberry cultivars. Goiffon et al. [36] reported pelargonidin- 3-arabinoside to be the third anthocyanin in two varieties (‘Senga sengana’ and ‘El santa’) instead of pelargonidin-3-rutinosied. According to Tamura et al. [37] Pelargonidin-3-malonylglucoside is one of the main anthocyanins in strawberry [32].

In general, pelargonidin 3-O-glucoside provides a bright red color to strawberries, whereas cyanidin 3-O-glucoside provides a darker red color [38]. Thus, the consumer preference over time for a bright red color has indirectly led to the selection of strawberry cultivars with pelargonidin 3-O-glucoside as the major anthocyanidin form, representing 70–90% of the total anthocyanins regardless of genetic and environmental factors [2].

However, in our experiment with different extraction buffers and three sample types (fresh, frozen and freeze-dried), Delphinidin (25) was the main anthocyanin, present in all extraction methods and sample types. Neder-Suárez [39] expressed that extraction methods significantly affect Cyanidin-3-glucoside content and could similarly affect other anthocyanins. Ghassempour et al. [18] also discovered that different extraction methods (organic solvent, ultrasound and microwave assisted) extracted different anthocyanin profiles from grape skin in their experiment. Malvidin-3-glucoside was the major compound from the organic solvent method, whereas in ultrasound and microwave assisted methods, other compounds such as malvidin-(6-coumaroyl)-3-glucoside, petunidin-(6-coumaroyl)-3-glucoside, and malvidin-(6-caffeoyl)-3-glucoside were dominant. We also identified different anthocyanins as the major compound in different extraction methods. Pelargonidin was the major compound in chloroform-methanol method suggested by Solovchenko et al. [5] (pH of 3.0), while delphinidin was dominant in other methods (pH between 1.0–2.0). Similarly, da Silva et al. [32] identified pelargonidin as the main anthocyanin in most strawberry cultivars tested. The observed behavior was the result of different extracting mechanisms or pH involved in the processes as suggested by Ghassempour et al. [18].

## 5. Conclusions

It is well documented that several factors during anthocyanin extraction affect the yield and profile of the extracted anthocyanins. Acidified solvents with chloroform-methanol have higher anthocyanin contents than water-based buffers. Methanol-water based buffers also perform better than methanol alone. Water-based buffers, such as pH differential buffer, have a lower total anthocyanin content, simply because methanol and chloroform are able to represent different anthocyanins into the extract that water alone cannot. Freeze-dried strawberries had higher anthocyanin contents compared to fresh or frozen samples.

Based on the results of this experiment, water-based extracts have maximum absorbance at lower wavelength (498 nm), methanol at mid-range (508 nm) and chloroform at higher wavelength (530 nm). Also, pelargonin had lower maximum absorbance (508 nm) than cyanidin-3-o-glucoside (530 nm).

Chloroform-methanol solvent extracted pelargonidin as the main anthocyanin, while other solvents (methanol, water) extracted delphinidin as their main anthocyanin. This effect could be due to higher pH (about 3.0) in the chloroform-methanol solvent compared to lower pH of 1.0 to 2.0 in other solvents.

Therefore, chloroform-methanol and methanol-water solvents are the best solvents for extracting anthocyanins from strawberries. However, if comparative studies of a large number of samples are needed, the pH differential method is a fast and reasonably reliable method of anthocyanin assessment in strawberries.

## Figures and Tables

**Table 1:** Total anthocyanin content of fresh, frozen and freeze-dried strawberry puree measured by organic solvent and pH differential methods.

**Method Tested**	**Anthocyanin Concentration (A/gFW)**	SD *
Clf-Methanol (Neff & Chory [7] ^(4)^	9.4 a	0.7
Methanol (Lindoo and Caldwell [6] ^(3)^	9.3 a	0.6
Clf-Methanol, Solovchenko et al. [5] ^(1)^	8.8 a	2.0
Methanol (Solovchenko et al. [5] ^(2)^	8.7 b	1.5
pH differential (Lee et al. [10] ^(5)^	6.5 c	0.4
Combined (Gauche et al. [11] ^(6)^	4.9 d	1.0
LSD	0.5	
**Sample type**	**Anthocyanin concentration (A/gFW)**	SD *
Freeze-dried	8.6 a	2.0
Frozen	7.9 b	2.1
Fresh	7.9 b	2.3
LSD	0.3	

Abbreviations: * SD, standard deviation; A, absorbance; C3G, Cyanidin-3-glucoside used as internal standard, Superscript numbers in the brackets refer to the method number; Six methods were tested: (1) and (2) methanol method by Solovchenko et al. [5] with and without chloroform (Clf) respectively, (3) methanol method by Lindoo and Caldwell [6], (4) chloroform(Clf)-methanol method by Neff & Chory [7], (5) pH differential method by Lee et al. [10], and (6) combination of methanol and pH differential methods by Gauche et al. [11]. This formula (*A*_530_ − 0.3*A*_657_ × 20)/1 was used to calculate total anthocyanin content for methods 1 to 4 and ((*A*_520_ − *A*_700_)pH1 − (*A*_520_ − *A*_700_)pH4.5 × 20)/1 for pH differential and combined methods. Different letters in each column were significantly different at *p* ≤ 0.05.

**Table 2:** The two main anthocyanins in the profile extracted by different methods from three sample types and identified by ultra high liquid chromatography- electrospray ionization tandem mass spectrometry (UHPLC-ESI-MS). The pH of the extract, the compound name, formula, retention time and charge/mass ratio is presented.

Method	Sample Type	pH	Compound Name	Formula	RT (Min)	m/z
	Fresh	3.02	Pelargonidin Petunidin	C_15_H_11_O_5_ C_16_H_13_O_7_	10.04 16.84	271.06 317.06
Clf-Methanol (Solovchenko) ^(1)^	Frozen	3.65	Pelargonidin Cyanidin	C_15_H_11_O_5_ C_15_H_11_O_6_	10.03 16.84	271.06 287.05
	Freeze-dried	3.024	Pelargonidin Cyanidin	C_15_H_11_O_5_ C_15_H_11_O_6_	10.04 16.83	271.06 287.05
	Fresh	2.21	Delphinidin Cyanidin	C_15_H_11_O_7_ C_15_H_11_O_6_	12.98 16.82	303.05 287.05
Methanol (Solovchenko) ^(2)^	Frozen	1.70	Delphinidin Cyanidin	C_15_H_11_O_7_ C_15_H_11_O_6_	12.98 16.81	303.05 287.05
	Freeze-dried	1.06	Delphinidin Pelargonidin	C_15_H_11_O_7_ C_15_H_11_O_5_	12.99 10.04	303.05 271.06
	Fresh	1.20	Delphinidin Cyanidin-3-O-galactoside	C_15_H_11_O_7_ C_21_H_21_O_11_	12.99 7.09	303.05 449.11
Methanol (Lindoo) ^(3)^	Frozen	1.23	Delphinidin Cyanidin-3-O-galactoside	C_15_H_11_O_7_ C_21_H_21_O_11_	12.99 7.08	303.05 449.11
	Freeze-dried	1.17	Delphinidin Cyanidin-3-O-galactoside	C_15_H_11_O_7_ C_21_H_21_O_11_	12.99 7.04	303.05 449.11
	Fresh	1.73	Delphinidin Malvidin-3-O-arabinoside	C_15_H_11_O_7_ C_22_H_23_O_11_	12.97 7.38	303.05 463.12
Clf-methanol (Neff) ^(4)^	Frozen	1.70	Delphinidin Pelargonidin-3-O-sophoroside-5-O-glucoside	C_15_H_11_O_7_ C_33_H_41_O_20_	12.98 8.82	303.05 757.22
	Freeze-dried	1.63	Delphinidin Pelargonidin	C_15_H_11_O_7_ C_15_H_11_O_5_	12.96 10.08	303.05 271.06
	Fresh	1.67	Delphinidin Pelargonidin	C_15_H_11_O_7_ C_15_H_11_O_5_	12.99 10.06	303.05 271.06
pH differential (Lee) ^(5)^	Frozen	1.25	Delphinidin Pelargonidin-3-O-sophoroside-5-O-glucoside	C_15_H_11_O_7_ C_33_H_41_O_20_	12.99 8.83	303.05 757.22
	Freeze-dried	1.26	Delphinidin Pelargonidin-3-O-sophoroside-5-O-glucoside	C_15_H_11_O_7_ C_33_H_41_O_20_	13 8.84	303.05 757.22
	Fresh	1.63	Delphinidin-3,5-O-diglucoside Delphinidin	C_27_H_31_O_17_ C_15_H_11_O_7_	1.1 12.98	627.16 303.05
Combined (Gauche) ^(6)^	Frozen	1.32	Delphinidin Delphinidin-3,5-O-diglucoside	C_15_H_11_O_7_ C_27_H_31_O_17_	12.98 1.11	303.05 627.16
	Freeze-dried	1.30	Delphinidin Delphinidin-3,5-O-diglucoside	C_15_H_11_O_7_ C_27_H_31_O_17_	12.98 1.1	303.05 627.16

Numbers (superscript) in the brackets refer to the method number. Abbreviations: Six methods were tested: (1) and (2) methanol method by Solovchenko et al. [5] with and without chloroform respectively, (3) methanol method by Lindoo and Caldwell [6], (4) chloroform-methanol method by Neff & Chory [7], (5) pH differential method by Lee et al. [10], (pH 1.0), and (6) combination of methanol and pH differential methods by Gauche et al. [11], step 2, (pH 1.0).

## Data Availability

Data is contained within the article, and will be provided on request.
